# Chladni Figures in Modal Analysis of a Double-Panel Structure

**DOI:** 10.3390/s20154084

**Published:** 2020-07-22

**Authors:** Jaroslaw Rzepecki, Anna Chraponska, Sebastian Budzan, Chukwuemeke William Isaac, Krzysztof Mazur, Marek Pawelczyk

**Affiliations:** Silesian University of Technology, Department of Measurements and Control Systems, Akademicka 16, 44-100 Gliwice, Poland; anna.chraponska@polsl.pl (A.C.); sebastian.budzan@polsl.pl (S.B.); chukwuemeke.william.isaac@polsl.pl (C.W.I.); krzysztof.jan.mazur@polsl.pl (K.M.); marek.pawelczyk@polsl.pl (M.P.)

**Keywords:** modal analysis, Chladni figures, double-panel structure, image enhancement, rigid device casing

## Abstract

Analysis of the structural vibration, under the sound excitation is an important part of the quality assurance during the design process of devices. One of the most commonly used method is Laser Doppler Vibrometry (LDV). However, under the rapid fluctuations of temperature, structural resonances are shifted into the other frequencies. In such situation LDV method may be inconvenient, due to the scanning time. In this paper the authors proposed Chladni figures to modal analysis of the double-panel structure, excited by the loudspeaker enclosed inside the casing with a rigid frame. Double-panel structure has been proven to be particularly useful for noise and vibration reduction applications. Vision images, obtained during the experiments are converted to binary patterns, using GLCM matrix, and compared with simulations performed in ANSYS.

## 1. Introduction

Noise exposure is a common issue, which may significantly annoy or even result in irreversible hearing loss of workers and people using noisy appliances in their everyday life [1]. Both noise and vibration have an impact on humans. Low-frequency vibration, specific to the machine operator’s profession, is especially harmful and causes decrease of work efficiency [2]. Vibrations in the form of shocks have dangerous impact on humans in vehicles, as resonant frequencies of the human body may be excited [3]. The aim of contemporary researchers is to minimize noise and vibration pollution in the human environment by improvement of the materials and methods.

Therefore, to reduce noise and vibration, three main groups of methods may be employed, i.e., active, semi-active or passive. In the traditional passive noise reduction techniques, absorptive materials or vibration absorbers may be used [4]. However, these techniques are ineffective at low frequencies [5], which is wider explained in [6,7]. In active methods, the goal of noise and vibration reduction may be achieved by external activation of sources in vibroacoustic control systems used in different ways [8,9,10]. In semi-active methods, specific materials or elements are used to modify on demand properties of the structure in such a way that vibration or noise propagation is reduced. As the industrial devices generate mainly low-frequency noise [11], and the semi-active and active methods are the most effective at low frequencies [10], the frequency range of interest in this research is limited to 500 Hz.

One of the approaches to globally reduce noise and vibration may be a casing, which encloses a noise-generating device. The authors previously examined the active [12] and semi-active [13,14] approaches to reduce noise or vibration with the use of light-weight and rigid casings. Novel improvements are being developed to enhance the effect of noise and vibration reduction.

In this research, a rigid casing [15] is under investigation. Such casing consists of a heavy frame with walls built of single- or double-panels, mounted on the frame. The plates considered in this paper are thin, which means their thickness is small compared to the plate’s lateral dimensions [16]. One of rigid casing walls is a double-panel structure, consisting of two steel plates placed at the distance of 50 mm between them. Each plate is attached to a heavy rigid frame with the use of 20 screws, hence fully clamped boundary conditions are assumed. In the examined approach, the semi-active and passive modifications of the double-panel structure are provided. The double-panel structure is modified using solenoids or neodymium magnets as the couplings between plates.

Double-panel structures have been of special interest nowadays because of good sound insulation they provide [4]. The design of such structure is important in many industries like automotive, aerospace [17], buildings [18], and the production of surface ships and submarines [19]. Since many years, the sound transmission through double walls has been modelled and analyzed [20], along with measurements. Many factors, which influence the propagation of sound waves through the plates in general, may be taken into account [21]: velocity, pressure, density, temperature, viscosity, bulk viscosity, gas constant, mean density, thermal conductivity, and specific heat at constant pressure and time. In the case of the double-panel structure, many parameters are involved in the transmission of sound wave, e.g., mass, density, Poisson ratio, Young’s modulus, the material properties, and the type of the source [22]. Moreover, in the double-panel structures, change of temperature also has an impact on natural frequencies, as they decrease with the increase of temperature, and the first resonance is the most sensitive to temperature load [23]. A double-panel structure is characterized by the mass-air-mass resonance, where plates of the structure move in the opposite phase [24]. It is beneficial to locate the frequency of the mass-air-mass resonance below the lower limit of the noise frequency range, because the sound transmission loss of a double-panel structure increases above the mass-air-mass resonant frequency [22]. Hence, the acoustic performance of the double-panel structure is also increased. In the double-panel structures, active noise control may be used to improve its sound transmission loss [25]. Moreover, double-panel structures may be sandwiched with interlayers and absorbing materials to increase their sound insulation performance [26]. Such structures are characterized by low weight and high strength [26]. Sandwich periodic structures are also investigated as they may provide both reduction of vibration and sound radiation [27].

Modeling of the double-panel structure is a complex task, as the vibro-acoustic interactions between the vibrating plates and the fluid in the cavity between the panels and in the interior of the casing itself have to be taken into account [28], along with the other factors mentioned above. In this research, numerical modeling with the use of ANSYS software was performed to simulate modal response of the double-panel structure under the excitation of a narrowband noise.

Modal properties of the plates may be analyzed with the use of many methods, e.g., with the use of Laser Doppler Vibrometry (LDV) [29]. However, if rapid temperature fluctuations of the structure are observed, they have an impact on resonances. Plates that are subject to spatial changes of temperature, undergo buckling, which causes mode shifting [30]. Hence, there is a need to introduce a different approach to modal analysis of the double-panel structure. In this paper, the authors propose to analyse the modified double-panel structure with the use of Chladni figures method and further advanced image processing, as well as a finite element numerical verification approach with the use of ANSYS software.

E.F.F. Chladni stated that shape of the transverse motion of a plate may be expressed by curved surfaces, while motionless lines are called nodal lines [31]. Chladni invented a method to visualize nodal lines by spreading a bit of sand on the plate’s surface and putting the plate into a motion, while one or more points are held immobile [31]. The vibration of the plate causes sand to move and accumulate on the nodal lines. However, Chladni’s ideas are applied commonly in the production of acoustic instruments, and they have not been thoroughly examined in the field of noise reduction research [32]. Chladni patterns have been mainly used to assess material constants of the single orthotropic plates [33] in recent years. They have been also applied to investigate the influence of surrounding fluid on the modal response of a single plate [34] and on manipulation of the particles’ motion [35]. Recently, this method has been compared with impulse hammer in modal analysis of a single simply-supported plate [36]. Another approach is determination of the Chladni figures by using optical methods [37].

In the Chladni experiment, it is necessary to spread a sand which is not too fine, to prevent it from attaching to the surface of the plate. In the classic approach, an excitation source is connected directly to a vibrating panel. In the case of the double-panel structure employed in this research, the noise source is not coupled with the panels. A loudspeaker is placed in the casing’s interior, with its cone facing the incident plate of the double-panel structure, thereby, causing it to vibrate. Both the fluid-structure and mechanical interactions between the plates cause the whole structure to vibrate. Sufficient level of excitation signal ensures that the amplitude of outer plate vibration is high enough, hence, sand is able to accumulate on the nodal lines, allowing to observe modal shapes. Experimental results are compared with the numerical simulations of the double-panel structure obtained with the use of ANSYS software. The detailed computational analysis of the examined system is crucial for the verification of experimental results [38]. The results show that Chladni figures method can be useful tool in modal analysis of the horizontally arranged, double-panel structures. In a spaces with rapid temperature fluctuations the use of LDV may not be reliable method, in opposite to Chladni experiment. However, the authors’ suggestion is to use this method as a preliminary, overall assessment of the mode shapes. Moreover, it is required to ensure proper object illumination by a lighting source. For improvement of the quality of the results, the obtainted images can be additionally processed by dedicated vision algorithm or fused with the data, acquired using another method, e.g., LDV.

The paper is organised as follows: Section 2 describes the numerical modeling of investigated double-panel structure. Also, the main assumptions and laboratory setup are presented. In Section 3, the considered cases and both of preliminary and main experiments are described. Also, the experimental results are discussed. Section 4 gives a summary and conclusion of the research presented in this paper. It contains main observations and ideas about potential opportunities and their application in practice.

## 2. Materials and Methods

### 2.1. Numerical Modeling of the Double-Panel Structure

**Figure 1:**
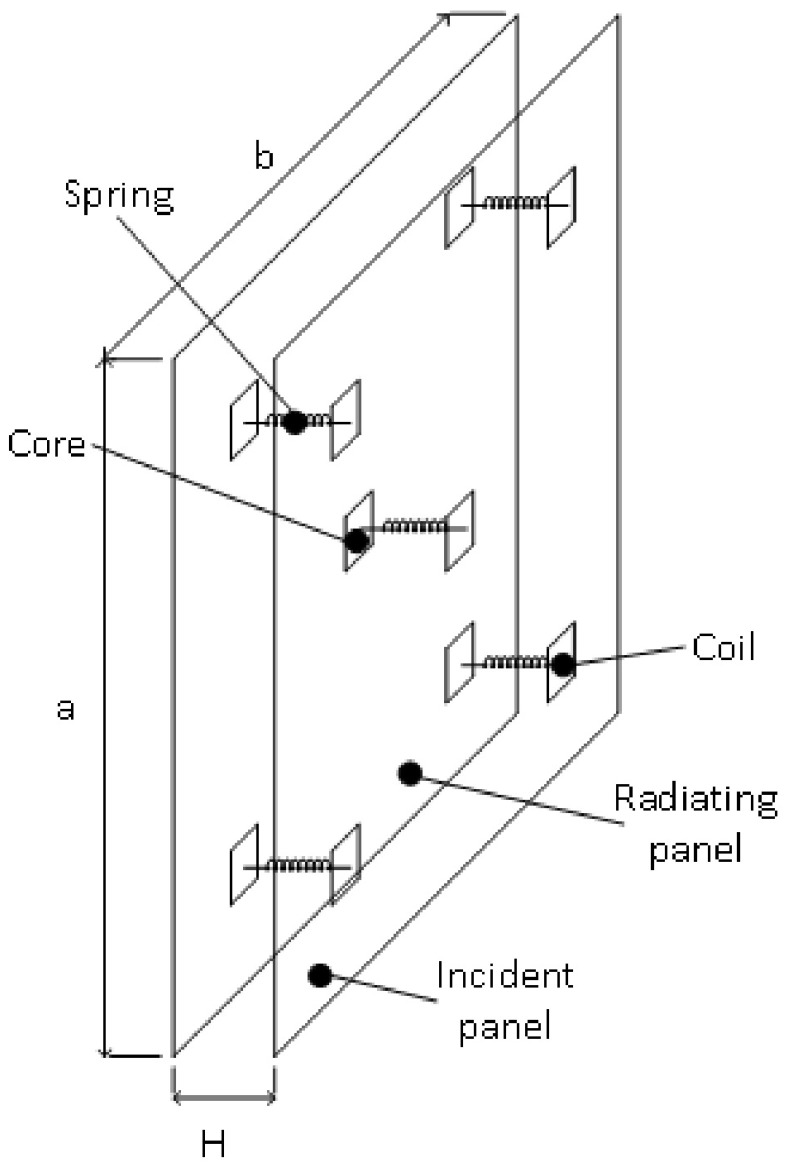
Scheme of double panel structure under mass-spring-mass vibro-acoustic excitation.

The finite double-panel structure (DPS) under consideration as shown in Figure 1 is made from a structural steel material, having equal length *a* = 420 mm, width *b* = 420 mm and thicknesses $h_{1}$ = 0.5 mm (for radiating plate) and $h_{2}$ = 0.6 mm (for incident plate). The DPS is immersed into an acoustic medium where a normal sound wave from a loudspeaker is used to excite the double panels filled with air of density $\rho_{a}$ = 1.225 kg/m${}^{3}$ and speed of sound $c_{a}$ = 346 m/s. Fully clamped boundary conditions are assumed and each of the panels is separated by a cavity depth of *H* = 50 mm. The density, Young modulus and Poisson ratio of the panels are 7850 kg/m${}^{3}$, 200 GPa and 0.3, respectively. The finite element software used for modelling the DPS is ANSYS Act-2019. For each panel, a 21 × 21 finite element discretization was adopted. Also, the acoustic air enclosure is discretized using an element size of 21.0 mm. The discretization of the structural panel and enclosure were achieved using SOLID 186 element type. The total number of elements obtained by the two panels was 79,163 while that of the nodes was 88,892. A distributed sparse matrix direct solver was used to solve the system of equations of the finite elements. Exemplary analytical modeling of the DPS is presented in Xin et al. [39].

**Figure 2:**
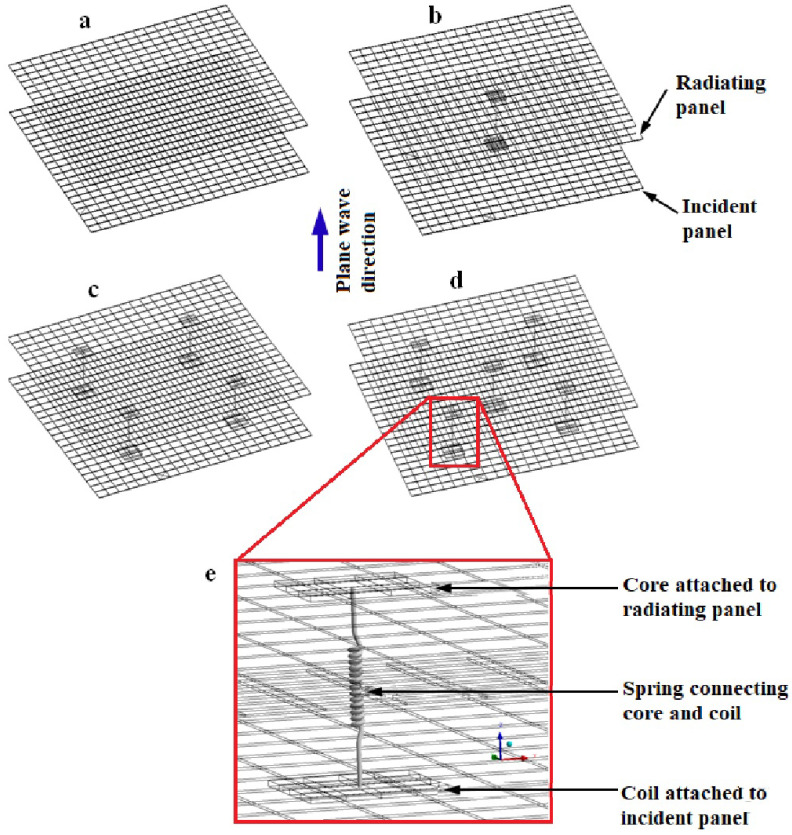
Undeformed mesh of DPS showing core, coil and spring connections: (**a**) no spring, (**b**) one spring, (**c**) four springs, (**d**) five springs, (**e**) spring connecting nodes of core and coil.

**Figure 3:**
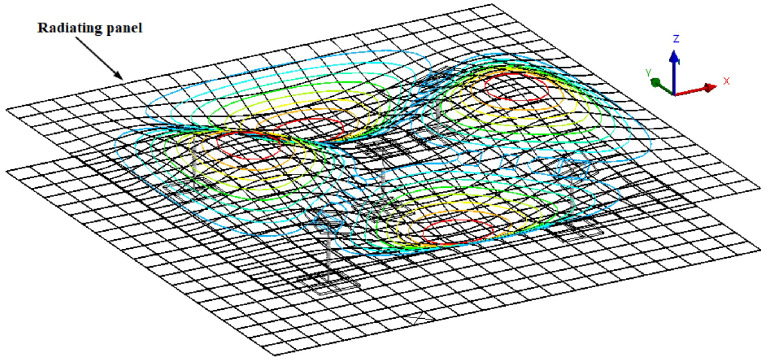
Deformed mesh of DPS with five-spring connections also showing isolines for the modal response of the radiating panel.

Coils and cores of solenoids are bonded to the inner surfaces of the two panels. They are examined here as an interesting solution to modify response of the double-panel structure. The total mass of each coil is 0.025 kg and that of each core is 0.004 kg. Five coils and cores are bonded to the surface of the panels. A 4 × 4 finite elements discretization was used for both the core and the coil and they are manually bonded to their respective panels using surface-to-surface contact. The solenoid used in the experiment is modelled as spring in the FE simulation and there is already a spring prototype connection in the software. Springs of assumed stiffness $k_{e}$ = 20 N/m are connecting the central nodes of the coils and the cores. In this study, different spring connections as used in the experiment are modelled. For example, one-spring, four-spring and five-spring connections to the DPS are illustrated in Figure 2b–d, respectively. Figure 2e shows an enlarged view of the spring connected to the mesh of the coil and the core. The length of each spring is approximately 50 mm which corresponds to the depth of the acoustic cavity. During the modal analysis of the vibrating DPS, in the spring connection, a “none” preload was assigned with environmental temperature fixed at 22 °C. The acoustic excitation of the incident panel causes the coils and the connected springs to vibrate while the emitted incident sound wave passes through the acoustic cavity to the radiating panel. This, in turn, transmits the sound wave into the acoustic medium. Figure 3 shows the deformed mesh isolines for the modal response of the radiating panel resulting from acoustic excitation and vibration of the five-spring connections.

### 2.2. System Description

**Figure 4:**
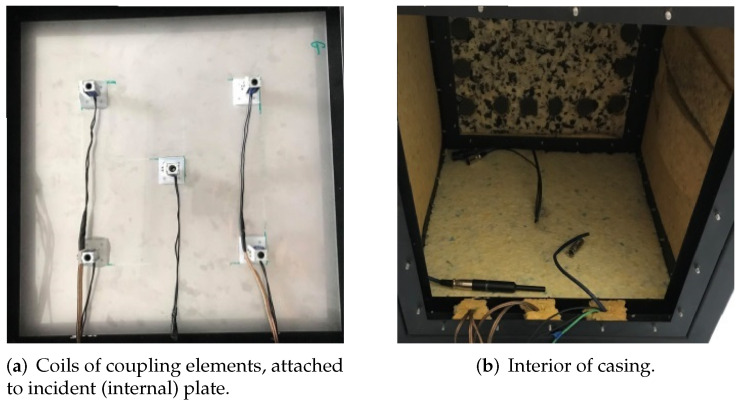
Investigated cubic casing with a rigid frame.

The double-panel structure, considered in this article is a modified top wall of the cubic casing with a rigid frame. Both incident and radiating plates are made from steel, and have the same dimensions. In the air cavity gap between the panels electromagnetic coupling elements are attached. The elements consist of coil, that induces electromagnetic force, when the current flows, and a ferromagnetic core, held inside the solenoid under impact of generated force. The coils are attached to the incident (internal) plate (Figure 4a), and the cores are mounted on the radiating (external) plate. Other, single panels of the casing were made from plywood with an additional bitumen layer, to enhance its acoustic insulation. As it was mentioned before, the inner side of the casing is sound insulated (Figure 4b). The panels are mounted by the metal frames with twenty screws, to provide boundary conditions similar to fully clamped.

**Figure 5:**
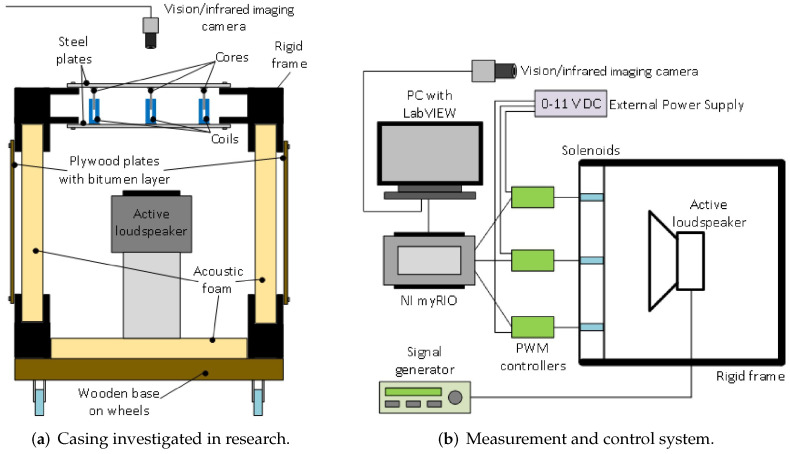
The schemes of laboratory setup.

The double-panel structure was excited to vibration by a tonal signal emitted by the active loudspeaker, enclosed in the investigated casing, at a distance of 100 mm from the incident plate (Figure 5a). The tonal signal was provided by external signal generator. Electromagnetic coupling elements were supplied by external power supply with the value of voltage between 0–11 V, controllable through the changing of duty cycle of PWM signal. In this experiment ON/OFF control algorithm was used, with constant (99 % to avoid coil ovearheating) value of duty cycle for ON state, and 0 % of duty cycle for OFF state. The PWM signal was provided by National Instruments myRIO platform, and the algorithm was implemented in LabVIEW graphical environment (Figure 5b). The main parameters of the solenoids provided in manufacturer’s documentation are presented in Table 1. The preliminary experiments were performed on the machine vision laboratory stand equipped with a 1624 × 1234 resolution color CCD camera, a wide 8 mm lens, 20 frames per second, and a diffused white light source. As part of the experiments, as a control tool for the vision system, an infrared imaging camera with a geometric resolution of 384 × 288 was used.

## 3. Results and Discussion

### 3.1. Preliminary Experiments

**Figure 6:**
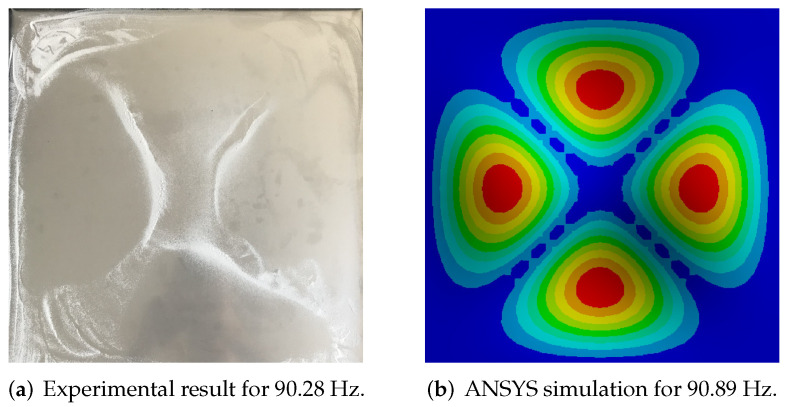
Selected modeshape.

The first experiment was performed for the one selected modeshape, at frequency 90.89 Hz. Following the Chladni’s experiment idea, the material used to observe the vibration of the radiation plate was corundum sand, due to its low viscosity properties. The shape obtained on the radiating plate was in line with results from the ANSYS simulation (Figure 6). The area with cumulated material is the nodal place, where the vibration amplitudes are the lowest. This preliminary experiment was performed before mounting of the solenoids, thus, there are no coupling elements between the plates and there are no additional masses.

**Figure 7:**
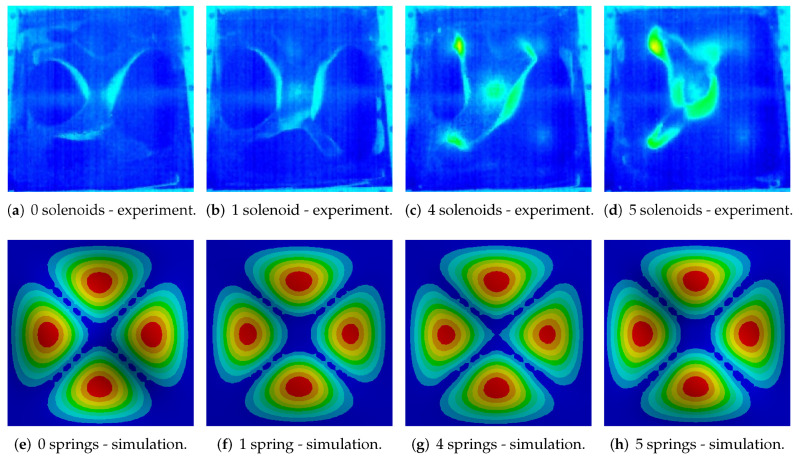
Experimental results for ON/OFF control algorithm compared with ANSYS simulations.

In the case of 2D/3D vision systems, an important element affecting the complexity of the image analysis and processing process is the appropriate selection of vision system elements. Analysis based on a 2D image is sufficient to obtain positive results, of course, while ensuring adequate image quality. It was obtained through the use of contrast powder, diffusion lighting, which was used to avoid local reflections on the tested plate. Importantly, it is initially covered with applied powder, while during experiments the powder naturally concentrates in local areas, which reveals the reflective surface of the plate. This feature was used in the proposed method for the detection of Chladni’s figures. Investigated modeshape was the same as previously, obtained for approximately 90 Hz (with slight fluctuations which are dependent on temperature and number of activated coupling elements). Four different scenarios were considered and the experiments were performed in the following order: all of the couplings in switch-off state (Figure 7a), single, central point activated (Figure 7b), four couplings in the corners activated (Figure 7c) and all solenoids activated (Figure 7d). The results were compared with the corresponding ANSYS simulations (Figure 7e–h). The IR images have attracted authors’ attention on two important issues: in every following image, temperature increase was observed and coupling force varied for every solenoid. This issue was clearly visible especially for the right-bottom area–the vibration amplitude was the highest in comparison with the other places of coupling and it could mean that the coupling force was lower than it should be.

Due to previously mentioned issues, the temperature fluctuations of the coupling element were investigated. During 30 s, which is approximate time of typical experiment, solenoid was supplied by voltage value similar to switch-on state from previous experiments. The temperature values are presented in Table 2.

During the time of experiment temperature fluctuations were significant, which resulted in resonance shifting to the lower part of frequency band and could be one of the reasons of deformation of the obtained shape.

**Figure 8:**
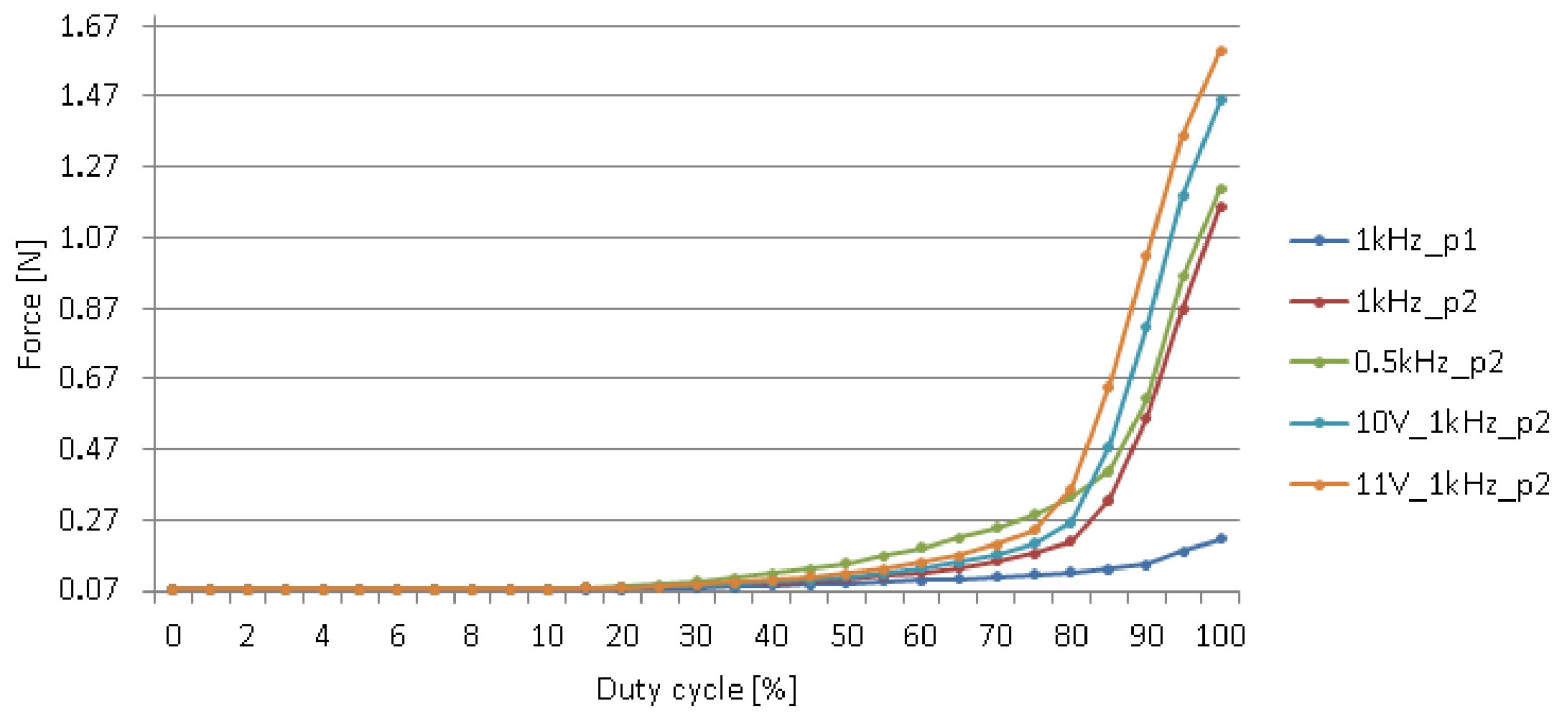
Comparison of the influence of solenoids parameters on generated force.

The next experiment was to verify influence of solenoids parameters on generated force, depending on duty cycle of PWM signal (Figure 8). Two different coil voltage values (10 and 11 V), two different PWM signal frequencies (0.5 and 1 kHz) and two different core positions (p1: 11 mm out of the coil and p2: 7 mm out of the coil) were considered.

**Figure 9:**
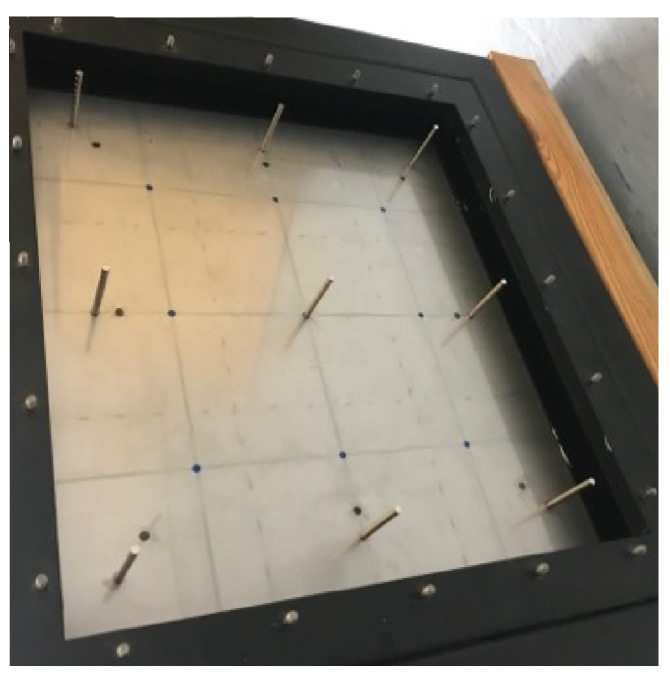
Neodymium magnets, mounted on the incident plate.

It was observed that the difference between generated forces depends on core’s position relative to coil’s geometrical center. Moreover, the lengths of solenoids cores were reduced to obtain the size that allows fit the elements between the plates. Slight inaccuracy of modification or montage process could change the force, generated by the solenoid. As it was not possible to provide identical accuracy of each solenoid’s assembly between the panels, electromagnetic elements were temporarily replaced by neodymium magnets [40] (Figure 9).

### 3.2. Image Based Analysis

**Figure 10:**
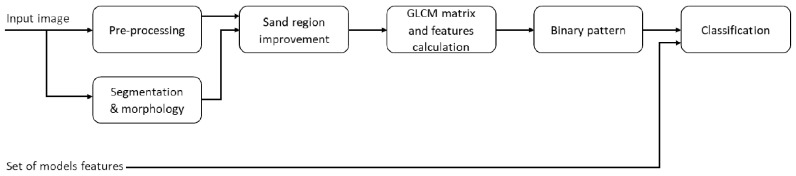
Scheme diagram of the proposed method.

Based on the computer vision information from the models presented in Figure 7, high-intensity areas that correspond to the areas separating individual Chladni figures are a characteristic feature. Given this, the authors decided to propose a solution that is based on the processing of digital images of the plate with sand. This concept is new, although some major machine vision devices are already used in the analysis of Chladni’s figures [41,42]. The proposed concept of the solution is based on processing a 2D digital image of Chladni’s figures into a binary pattern, which can then be analyzed as a string of 16, 64 or 256 elements. Each binary pattern element corresponds to a specific area of the image and describes its characteristics based on the Gray-Level Co-occurrence Matrix (GLCM). Each of these areas represented in the form of a binary pattern is distinctly different only to a specific number of solenoids and a specific frequency. Importantly, it is only by analyzing the interrelationships between areas that one can conclude about the appearance of a specific pattern of Chladni’s figure. The scheme diagram of the proposed method is presented in Figure 10.

**Figure 11:**
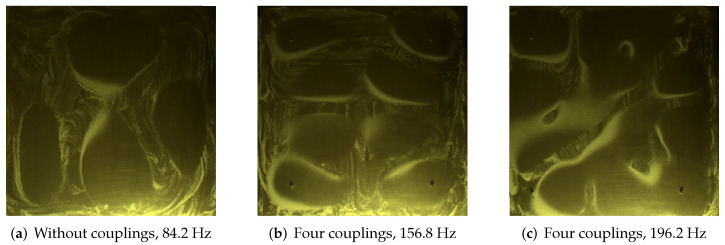
Samples of different mode shapes.

The input to the algorithm is a 2D digital image, however, due to its quality, especially low sand contrast on the surface of the plate, two-way processing was proposed. Its purpose is to extract the sand area as accurately as possible. Importantly, the sand is found on the entire surface of the tested plate and only in some places it accumulates depending on the number of solenoids and frequency. During the research, the authors also noticed a formation of the so-called conglomerates of sand grains, which is manifested by the appearance of clusters of smaller sand grains being a result of vibrations stick together, which could significantly affect the analysis of such an image. As can be seen in Figure 11, the plate was illuminated with a lightning source at an acute angle to show the entire surface of the plate and the individual grains of sand on it. This method is popular and used for detection of small-size objects, in the order of mm. On the other hand, the effect of light reflections in areas that are important to us from the point of view of assessing the shape of the figure were obtained. The consequence of the observed features is the introduction of pre-processing and segmentation in the method, which ultimately allows very good extraction of sand regions.

Taking into account previous observations, it is necessary to improve the contrast in the image as well as to bring out areas of sand. First, the pre-processing stage contains a contrast improving technique. Global enhancing methods improve the image contrast by extending a dynamic range of intensity using the histogram of the complete image. On the other hand, the local approaches use only local information inside each separated part of the image. In the recent literature there can be found numerous methods such as based on the bi-histogram equalization median plateau limit [43], a combination of Histogram Equalization (HE) and histogram clipping in exposure-based sub-image histogram equalization [44], also gradient-based local histogram equalization to preserve the image texture [45]. The authors decided to use HE, which changes the mean brightness of the input image to the middle level. The pixels in the image contain only information about sand localization, thus global HE is enough for numerous single objects located on the homogenous background. On the other hand, HE can increase the dark regions in the image without disruption inside individual regions.

**Figure 12:**
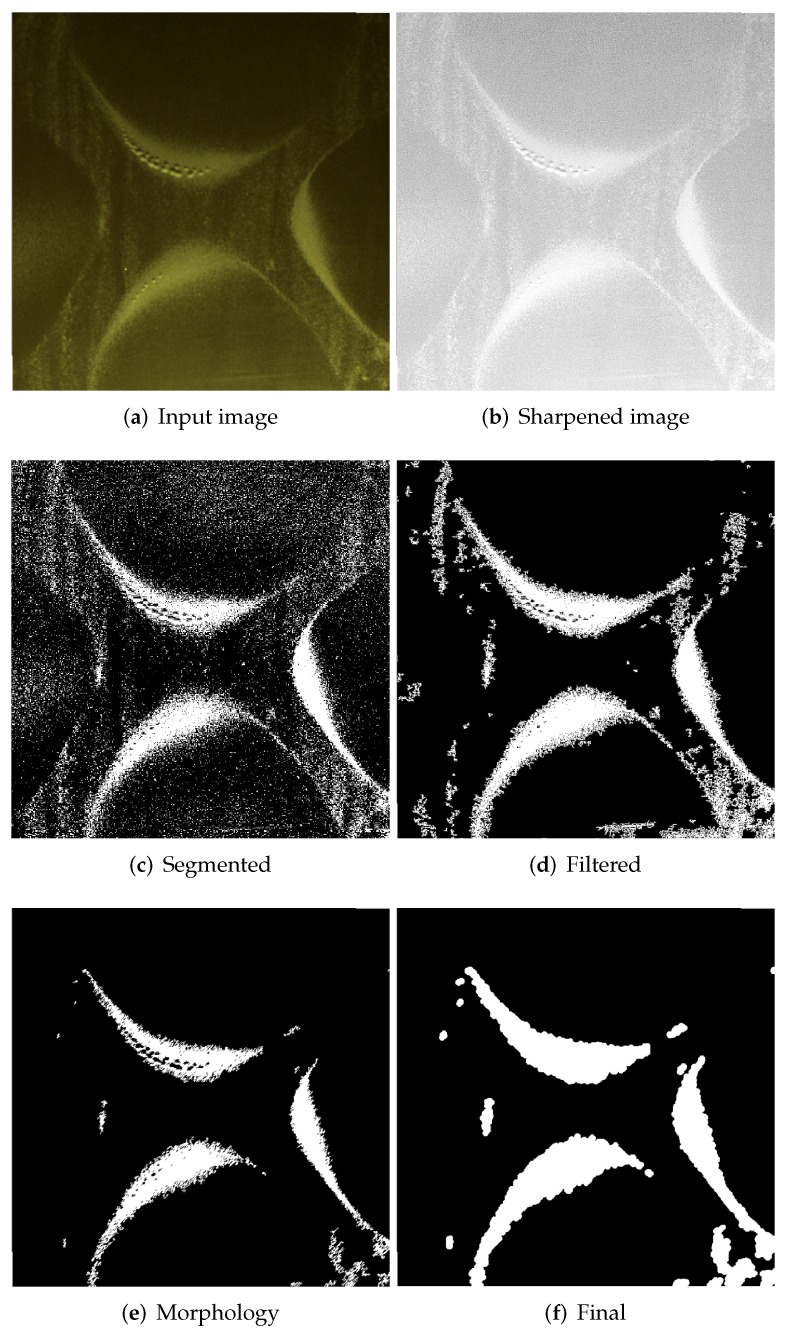
Samples of a mode shape at different stages of image.

The image after HE contains a significant amount of information that is unnecessary in the process of assessing the shape of Chladni’s figures, therefore, in parallel to the pre-processing, the authors decided to process the image, which is based on segmentation and morphology of all regions, and then to extract the sand region based on the image by combining images after HE and segmentation. Figure 12 presents the next steps to extract the sand regions: original image (Figure 12a), sharpened (Figure 12b), after segmentation (Figure 12c), after filtering small objects (Figure 12d), after initial morphological operations (Figure 12e), the final result (Figure 12f).

**Figure 13:**
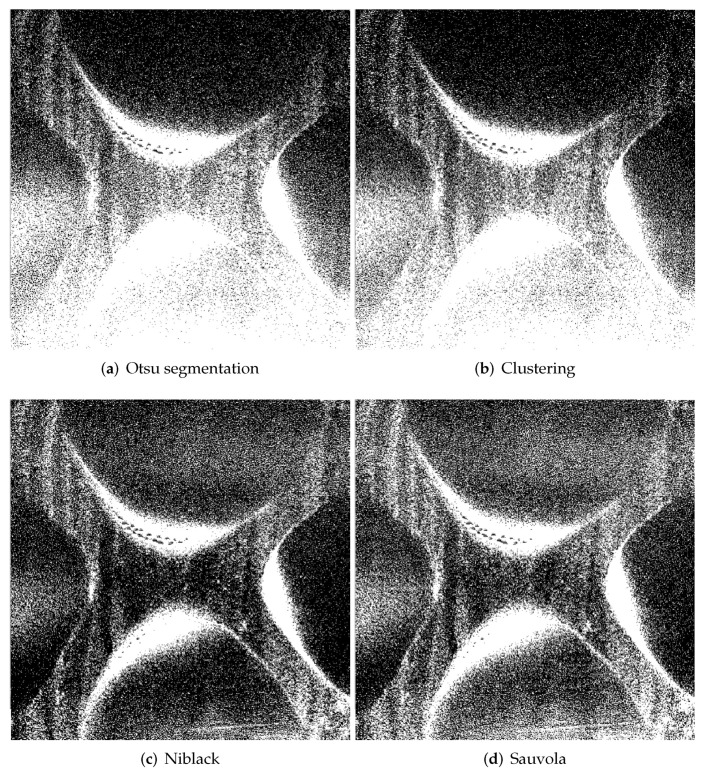
Results for different segmentation methods.

Description of the regions in the image is basically possible in two ways, namely edge-based and region-based approaches. The first method is used in situations, where the image is characterized by high contrast and objects appearing in it are easy to separate. Edge-based methods are adequate for detecting step changes in the images. Region-based methods are more suitable for flat differences between objects in the image, but separate objects must be homogeneous in luminance manner. Typically segmentation affects the final result by removing some pixels from the image which exceed some threshold value. This is a simple thresholding technique, which simply reduces or enlarges the segmented region by the extraneous pixels. This problem can be solved in two ways: local and global. Global segmentation methods generally divide regions in images into two classes such as object and background. The common method is based on the Otsu algorithm [46]. The method computes histogram and probabilities values for each intensity level of the image, which is called the threshold. By using global segmentation it is necessary to deal with false object boundaries by segmentation without correspondence to the real one edge, addition outside pixels, or removing pixels inside the region of interests. Other hand, the local methods are based on local information for each pixel or small region such as intensity or variance in the region. To the main local techniques, Niblack [47] and Sauvola [48] can be included. Some sample results of performed experiments with different methods are presented in Figure 13.

**Figure 14:**
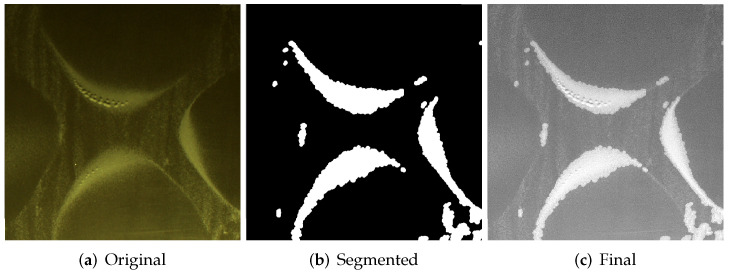
Final result of combination original image (**a**), segmented (**b**) and (**c**) Final.

Based on the experiments with different methods of segmentation the authors selected the Niblack method as more suitable for images acquired during this research. The proposed method is insensitive to lighting affecting the image and also retains pixels on the border of areas much better. Of course, the method generates a significant number of small-sized areas, but they are reduced in a further step, obtaining the image shown in Figure 14b. Next, both, segmented and pre-processed image is combined in a linear manner, in which large regions with sand will be increased, respectively small regions will be reduced. The final image (Figure 14c) with refined sand regions is then used to calculate the GLCM matrix and consequently GLCM features.

In the proposed solution, the authors rely on the analysis of independent, symmetrical local areas. The image is pre-divided into 16, 64 or 256 areas. In each of the areas, the GLCM matrix and such features as energy, homogeneity and contrast are calculated. The values of these features in the next step allow to specify a binary value (0/1) for the area. Finally, a matrix of e.g., 256 binary values, which represent all areas in the image, and are a unique binary formula, was obtained. Similarly, using the same image processing method, binary patterns for models from ANSYS were obtained. Then, based on the individual characteristics of these patterns, authors can classify a specific image from the experiment to the selected model for which its similarity is highest.

**Figure 15:**
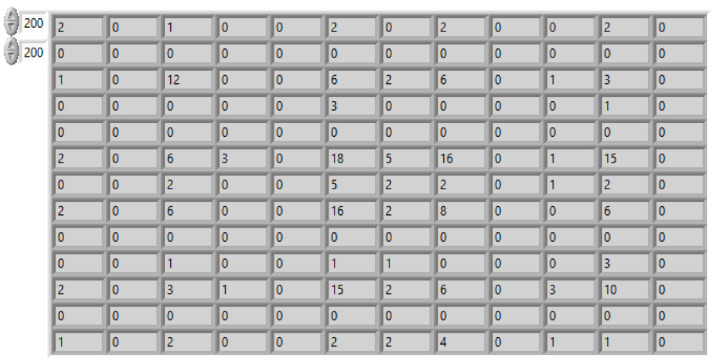
Part of the GLCM matrix for image in Figure 14c.

The GLCM matrix contains calculated results for all the transactions between intensities at specified positions relative to each other in the image (see Figure 15). The method yields information on how often a pixel with one gray-level value occurs either horizontally, vertically, diagonally bottom left to top right or diagonally top left to the bottom right to all the neighboring pixels with some other value. Consequently, the size of the GLCM matrix depends directly on the number of gray levels in the image. Importantly, the GLCM matrix values will be significantly different between large homogeneous sand areas and areas that contain sand in much smaller groups. For the first areas, the values in the matrix will be centered around the index values corresponding to high intensities, while for the second areas they will be more uniform. Haralick proposed 14 different textural features for different textural features in the image [49]. Based on the experiments three of them have been selected, i.e., energy, homogeneity and contrast. The energy is the sum of squares of values in the GLCM and should be high for images with high homogeneity. Image with constant value has energy equal to 1. The homogeneity feature is an opposite feature to the contrast, if the image contains homogeneous regions, the value should be closer to 1. All three features values in the binary values calculation process regarding a simple rule were used, so that the difference between calculated values and nominal for ideal flat and constant image region should be less than 5%.

**Figure 16:**
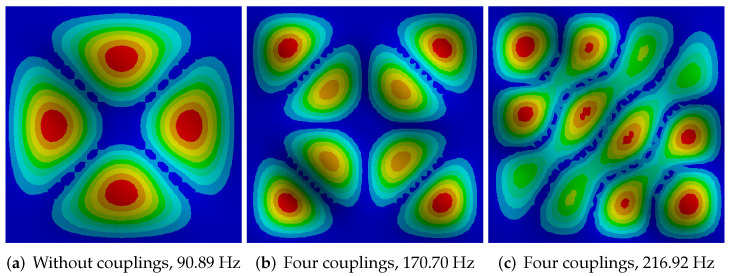
ANSYS models with different mode shapes, corresponding to the experimental results (Figure 11).

**Figure 17:**
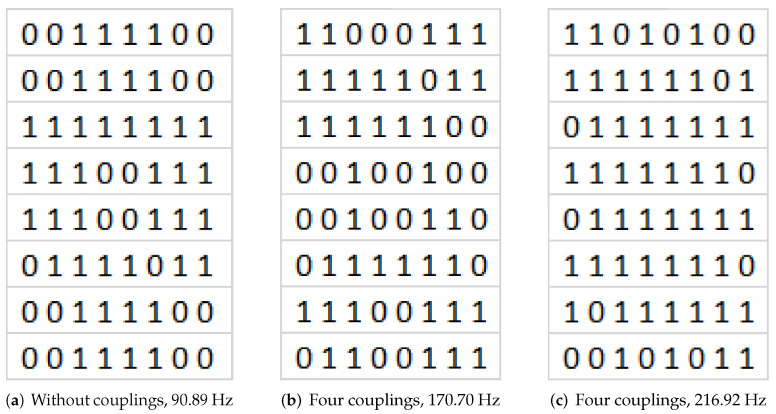
Calculated binary patterns (64 regions) for presented models (Figure 16).

Figure 16 shows images from ANSYS with varying degree of shape complexity. For this reason, the authors examined the possibility of dividing the image into 16/64/256 areas firstly, because for less complex shapes the proposed concept for calculating the binary pattern works very well (Figure 16a), while complex figures already require the division of the image into 256 regions due to the degree of detail. This situation is very well visible in Figure 16c, where a part of the regions is represented by the value 1 instead of 0, and this is where the amplitude is the smallest and the distance between the maximum amplitudes is insignificant. Results of binary patterns calculation with proposed method are presented in Figure 17, respectively to the Figure 16.

**Figure 18:**
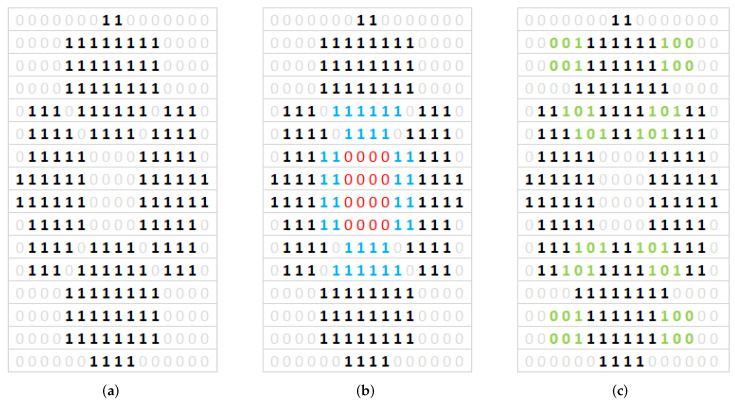
Calculated binary patterns (256 regions) for presented models (Figure 16a) with use of proposed method. (**a**) generated binary pattern, (**b**) marked first cascade elements, (**c**) marked second cascade elements.

Considering the above observations, the authors decided to base on 256 regions, because even for common shapes, the differences in shape between the figures for different numbers of solenoids (Figure 5) are slight and their detection is possible at sufficiently high resolution binary pattern. For 64 regions some details of the models are removed or deformed, which in consequence will affect the segmentation step. Examples of obtained binary pattern are presented in Figure 18. The calculated binary pattern can be used in the process of classifying images to one of the predefined groups, which is similar to the one, specified number of solenoids and frequency. The used classification is not based on a direct comparison of binary pattern elements to the nominal model, but on the examination only of selected groups of elements and their relationships, such as value, number and distance. The preliminary experiments in this issue were limited to developing a cascade rule-based system. In the first cascade, the number of possible classifier models is reduced, based on the degree of complexity of the shape, which is recognized by processing binary pattern elements in the center region of the image (red and blue elements in Figure 18b). As the output of this cascade, the similarity measure of processed shape to the models set is calculated. In the next cascade, a more detailed classification can be done with the use of predefined regions of the image. These regions represented by parts of binary patterns are unique for different model shapes. Examples of unique binary patterns elements can be seen in Figure 18c marked with green color. The proposed classification method depends strongly on the precision of the developed rules and number of the predefined models, what’s important especially for recognizing differences between similar in shape figures, but different in frequency and number of solenoids manner (see Figure 5). Moreover, the classification result is also influenced by the method of sand heap, which means that not all binary pattern elements during classification can be analyzed, but only those representing regions where the amplitude is the smallest, which is visible in the real image in the form of grain accumulation sand in the area, respectively in the model binary pattern will be the region of elements represented by 0.

**Figure 19:**
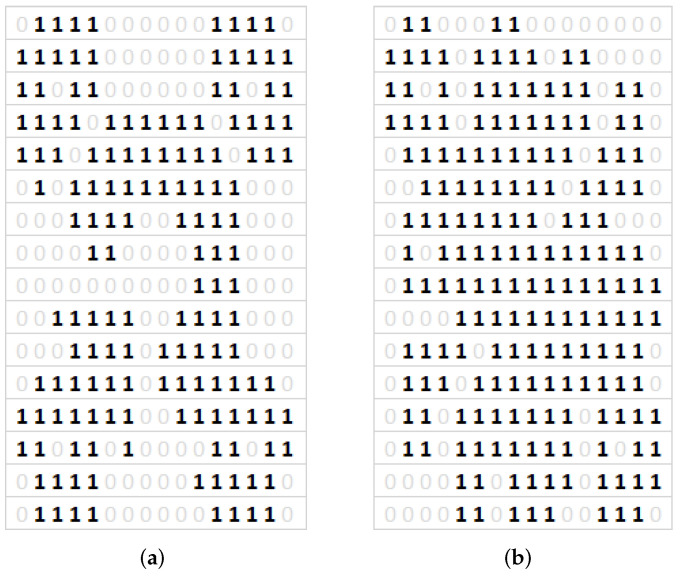
Binary pattern for complex Chladni’s figures from acquired images classified as (**a**) model Figure 16b, (**b**) model Figure 16c.

The preliminary experiments carried out are promising, as in the case of type Figure 18a Chladni’s figures we obtained the correct classification accuracy of 90%. The proposed rules and the method of creating a binary pattern effectively deal with image imperfections, such as the lack of some fragments of figures, which is especially visible in Figure 14 (right Chladni’s figure). However, for more complex Chladni figures such as Figure 19 classification level produce still correct result based on unique definition of region rules in satisfactory amount of samples, but with accuracy about 75%. Some imperfections in binary pattern, which can be seen especially in Figure 19b can be reduced by correct selection of regions–parts of the binary pattern which will be checked each time. The accuracy of the classification can be improved by more detailed rules, also by an adaptive weighting of the different combinations of binary patterns. Thus, the further work will be conducted on the automatic feature selection instead of the rule base created by the expert. The Current solution is sensitive for expert selection of features, which are taken into account with the same weight as others. Feature selection will be performed on Support Vector Machines with AdaBoost algorithm. Also, the method of region recognition will be improved with a combination of segmentation and edges of the regions.

## 4. Conclusions

In this paper, the authors investigated the well known Chladni figures method to obtain modeshapes of a plate in a different approach. In opposite to classical form, where a single panel is considered, the examined structure included two panels with the coupling elements between them. Moreover, there was no mechanical coupling between the structure and the excitation source, as in the classical approach, and different (fully clamped) boundary conditions were assumed. Additionally, the considered resonant frequencies were limited to 500 Hz. At the beginning, electromagnetic coupling elements were used. However, during the following experiments some issues were observed. Despite the fact that rapid fluctuations of temperature may result in a change of the resonant frequencies of double-panel structure, the short time of experiment allowed to observe mode shapes similar to numerical model outcome. On the other hand, modifications applied to solenoids construction affected differences between forces generated by those elements, what was confirmed by performed experiments. The most important modification was shortening of the core. The electromagnetic force attracting the ferromagnetic core is the sinusoidal function of distance from coil’s center, and even slight changes of the core strode can affect significant difference in holding force generated by the element. This is a serious issue, which eliminates the use of this kind of solenoids in such application. One of the most important requirements for the coupling elements is assurance of the same stiffnesses. Due to that conclusion, the next experiments were performed with the use of neodymium magnets, which provided sufficient stiffness of the couplings between the plates.

The experimental results lead the authors to a conclusion, that Chladni figures may be an interesting method for analysing modeshapes of the structures, under affection of rapid fluctuations of temperature, because of fast time of the experiment in comparison e.g., to LDV (when the high-resolution grid is assumed). The next advantage is an ability to observe slight amplitudes changes on the structure’s surface, which may not be possible in machine vision with the use of high-speed camera. The Chladni method does not require multi-channel data acquisition system, like in case of grid of accelerometers, mounted on the structures surface. Finally, in comparison to the acoustic methods (e.g., Near-Field Acoustical Holography) this method is robust to environmental noise. Despite of many advantages of the Chladni method, it is necessary to take into account its limitations: requirement of planar, horizontally placed and smooth surface of the structure, contact measurement and high sensitivity on air flow. The results prove that this method can be used also for more complicated structures, without necessity for coupling the investigated surface and source of excitation, in opposite to cases of typical use of the Chladni method.

## Figures and Tables

**Table 1:** The main parameters of solenoids.

Parameter	Value
Operating voltage	6 V
Operating current	0.3 A
Maximal force	5 N
Maximal voltage	12 V
Maximal current	1.5 A
Maximal stroke	10 mm

**Table 2:** Fluctuations of the solenoid’s temperature; voltage on coil: 11 V, current: 0.85 A.

Time [s]	Temperature [${}^{{}^{\circ}}$C]
0	22.1
5	25.9
10	29.6
15	32.6
20	35.9
25	39.8
30	41.5
